# Rationality over fashion and hype in drug design

**DOI:** 10.12688/f1000research.52676.1

**Published:** 2021-05-18

**Authors:** José L. Medina-Franco, Karina Martinez-Mayorga, Eli Fernández-de Gortari, Johannes Kirchmair, Jürgen Bajorath

**Affiliations:** 1 DIFACQUIM research group, Department of Pharmacy, School of Pharmacy, Universidad Nacional Autónoma de México, Mexico City, 04510, Mexico; 2 Instituto de Química, Universidad Nacional Autónoma de Mexico, Mexico City, 04510, Mexico; 3 Nanosafety Laboratory, International Iberian Nanotechnology Laboratory, Braga, 4715-330, Portugal; 4 Department of Pharmaceutical Sciences, Division of Pharmaceutical Chemistry, University of Vienna, Vienna, 1090, Austria; 5 Department of Life Science Informatics, B-IT, LIMES Program Unit Chemical Biology and Medicinal Chemistry, Rheinische Friedrich-Wilhelms-Universität, Bonn, D-53115, Germany

**Keywords:** artificial intelligence, computer-aided drug design, drug discovery, chemoinformatics, education, open science

## Abstract

The current hype associated with machine learning and artificial intelligence often confuses scientists and students and may lead to uncritical or inappropriate applications of computational approaches. Even the field of computer-aided drug design (CADD) is not an exception. The situation is ambivalent. On one hand, more scientists are becoming aware of the benefits of learning from available data and are beginning to derive predictive models before designing experiments. However, on the other hand, easy accessibility of *in silico *tools comes at the risk of using them as “black boxes” without sufficient expert knowledge, leading to widespread misconceptions and problems. For example, results of computations may be taken at face value as “nothing but the truth” and data visualization may be used only to generate “pretty and colorful pictures”. Computational experts might come to the rescue and help to re-direct such efforts, for example, by guiding interested novices to conduct meaningful data analysis, make scientifically sound predictions, and communicate the findings in a rigorous manner. However, this is not always ensured. This contribution aims to encourage investigators entering the CADD arena to obtain adequate computational training, communicate or collaborate with experts, and become aware of the fundamentals of computational methods and their given limitations, beyond the hype. By its very nature, this *Opinion* is partly subjective and we do not attempt to provide a comprehensive guide to the best practices of CADD; instead, we wish to stimulate an open discussion within the scientific community and advocate rational rather than fashion-driven use of computational methods. We take advantage of the open peer-review culture of *F1000Research* such that reviewers and interested readers may engage in this discussion and obtain credits for their candid personal views and comments. We hope that this open discussion forum will contribute to shaping the future practice of CADD.

## Computer-aided drug discovery

Computer-aided drug discovery (CADD) has become a key technology in drug discovery, providing guidance to experimentalists on which compounds and experiments to focus on next. The capacity of CADD has further increased by the development of powerful machine learning approaches, deep learning in particular. ^1^ In recent years, software for CADD has become more widely accessible. Today, a range of software packages are available that are open-source or free to use for academic research. ^2– 5^ Together with significant alterations of the scientific landscape induced by the COVID-19 pandemic and the ensuing re-orientation of some early-career but also established research groups towards the use of computational tools, this has boosted the use of CADD methods in particular in academic research environments. However, low-barrier access to computational tools and computing power increasingly leads to the use of CADD techniques also by scientists who have not received formal training on these methods. Many newcomers to CADD employ easy-to-use software without realizing the complexities involved and without being aware of the many potential pitfalls. Improper use of CADD techniques can have a contrary effect on research than intended. The risk of generating meaningless or false predictions is high. Flawed predictions can lead to dedicating significant resources to futile experiments. In particular, publishing invalid predictions can lead to error propagation, eventually resulting in a loss of confidence in CADD. Moreover, uncritical or naive use of artificial intelligence (AI) methods that are being heavily promoted in many research fields has similar negative effects, working against the credibility and acceptance of CADD as a scientific discipline.

Good practices to conduct and report studies in computational medicinal chemistry and chemoinformatics have been outlined in several articles. ^3, 6, 7^ Similarly, best practices in different stages involved in CADD have been discussed, for instance, in quantitative structure-activity relationship (QSAR) analysis, ^8^ data curation, ^9^ molecular docking ^10, 11^ and virtual screening. ^12^

In this contribution, we discuss common misconceptions and false expectations associated with CADD, especially in the AI era, and make recommendations on how to avoid common pitfalls when using CADD software. We aim to stimulate an open discussion within the community to help improve our perception and practice of CADD and contribute to shaping its future.

## CADD and related fields

Experts from various disciplines involved in drug discovery, such as chemical synthesis and biochemistry, are increasingly making use of computational tools to guide their experimental research and rationalize their observations. This is a positive trend, as developers of CADD tools have been aiming for a long time to make their software more widely accessible and intuitive to use. Prominent examples include web servers, and commercial, free and open-source software. But to develop further, it is of paramount importance to avoid confusion about the concepts and differences between the different areas in drug discovery such as molecular modeling, chemoinformatics, and theoretical chemistry. Conceptual and practical differences between these disciplines are clearly described in the literature. ^13, 14^ Importantly, theoretical disciplines are an integral part of CADD, establishing its scientific foundations. The ability to apply such approaches using software does by no means guarantee that reasonable research is carried out. Therefore, any conclusions or claims should be carefully considered.

## Common misconceptions and false perceptions when CADD is superficially viewed

The advent of more advanced computational tools, many of which are open source, freely accessible, and promoted as “easy-to-use,” also increases the widespread use of “buzz words,” and misconceptions among newcomers to CADD.

Table 1 gives examples of incorrect expressions and misconceptions frequently affecting students and researchers with little or no expertise in CADD. Readers and peer reviewers are welcome to comment openly on these points and modify or enrich the list according to their own experience.

**Table 1.** Examples of misconceptions versus the intended use accepted by experts in the field.

Misconceptions or misleading statements	Correct meaning
*Computational methods are fast and cheap, and in particular in the pandemic situation we can, and should, put experimental research on hold and turn to in silico methods.*	In silico studies may be conducted independently of experiments. In fact, theoretical approaches may address research questions that are beyond experimental accessibility. Ideally, computation and measurement are integrated, e.g. for the purpose of model validation and in applied research.
*Computational studies are fast and easy to conduct, and they always produce results.*	Depending on the research question, computational projects can in fact be resource-demanding and time-consuming. The fact that they “always” produce results does not mean that these results are “always” valid (this is certainly not the case). Use of any results without careful vetting is related to a significant risk of predictions being false or inaccurate.
*Purely computational studies have limited value and do not represent standalone projects.*	A purely computational study can be self-contained and comprehensive and may address research questions going beyond experimental accessibility.
*In multidisciplinary projects, experimental testing is difficult but computational studies are easy.*	Both computational and experimental work might be routine or challenging. The development and validation of new algorithms may well exceed the magnitude of experimental work.
*Computational analysis mostly contributes catchy pictures to publications and grant applications.*	If properly conducted, computational analysis can rationalize experimental observations and yield experimentally testable hypotheses.
*Machine learning and AI are the new standard for CADD.*	Machine learning is a part of AI and already has a long record of use in CADD. While being important to many types of predictions and enabling new applications, machine learning methods on their own have not revolutionized the field (yet).
*Molecular modeling and chemoinformatics are other terms for CADD.*	CADD covers various theoretical disciplines including molecular modeling, chemoinformatics, bioinformatics, theoretical chemistry, and machine learning.
*Molecular docking can be used to demonstrate ligand binding.*	Molecular docking approximates protein-ligand interactions and binding modes in a computational complex manner that only partly resembles physical binding events. Entropic effects in particular are only poorly considered by docking approaches.
*Rational drug design must incorporate a computational analysis.*	A drug can be rationally designed based on prior knowledge, experience and even causal intuition, without the need to employ a computer.
*Computational results are unbiased and thus most likely correct.*	Any computational analysis is affected by methodological limitations in accounting for the physical reality. Hence, results must be interpreted with caution and awareness of such limitations. It is ultimately the responsibility of the researcher to arrive at a scientifically sound and trustworthy interpretation of the results.
*Most computational techniques can be learned in a few hours of hands-on workshops.*	How to execute a software might be learned rapidly, but understanding the theory behind a computational approach and gaining the experience essential to the correct interpretation of the relevance, meaning and reliability of predictions can be demanding and take a significant amount of time. Without a firm grasp of the underlying theoretical foundations, computational exercises may only lead to appealing but meaningless illustrations.
*In contrast to reagents for organic synthesis, no “purification” is needed for the input of computational approaches.*	Data curation is of fundamental importance to CADD. Without proper curation of the input data, no meaningful predictions will be possible. The role of data curation in CADD equals that of experimental preparation and reagent purification in chemical synthesis.
*In contrast to reaction mechanisms in organic synthesis, understanding of how an algorithm works is not required in order to produce predictions.*	Understanding algorithms is as important as understanding experimental approaches.
*In contrast to a biochemical assay, controls are not required in a computational analysis.*	Including controls into the calculations is an indispensable part of any scientifically sound computational study.
*There are no computational “experiments”.*	An inferior computational analysis will produce results meeting our pre-formed expectations. A properly designed computational study will unambiguously address a question or hypothesis for which we have no answer to yet. This represents, in its best sense, an “in silico experiment”.

## When is a study “complete”?

Traditionally, there is a widespread belief in experimental sciences that experimental results represent reality, disregarding the different way in which natural phenomena can be represented and perceived and the relativity associated with varying representations. This ideological attitude works against “out of the box”, hinders intellectual progress, and indirectly de-values scientific disciplines such as CADD. With the rise of AI as one of the most heavily promoted approaches in contemporary society, the academic community has been encouraged to redirect its attention to computational tools to enhance its research impact and appeal. Nevertheless, unconditional trust in “experimental reality” reduces CADD to a “tool provider” and does not regard it as an independent scientific discipline in its own right. Consequently, computational models are often used without the necessary theoretical understanding and the rigor needed to apply them systematically.

In the authors’ opinion, one of the first requirements a new computational practitioner needs to address is realizing that both experimental and computational results are constrained by the model or experimental framework applied to determine them and, in no case, an absolute account of reality. Among medicinal chemists, there is the frequent misconception that purely theoretical or computational studies are in principle “incomplete” because there are no “real” experiments. However, such views require reconsideration and correction, as pointed out above. Rigorous computational studies answer questions that are difficult to address without “in silico experiments”. As such, they are comprehensive and self-contained, regardless of whether the computational approach has led to experiments. “Complete” computational investigations are often consistent with prior experimental observations, but may also chart new scientific territory. Of course, new computational insights leading to experimental work trigger interdisciplinary research. This is a noted strength of CADD, if conducted properly. However, there are misconceptions at interfaces between computation and experiment. For instance, a common malpractice is trying to replace enzymatic inhibition assays with predictions based on molecular docking or dynamics simulations. Another misunderstanding is that black box predictions from machine learning would represent a form of “alchemy”. What we cannot understand is not necessarily incorrect and may have value. The catch is that we are left with making decisions in such cases, for example, about new experiments that go beyond our reasoning and hence require trust in computational work and prior experience. It is also false to believe that AI in its current state would provide solutions to questions that replace our judgment capacity. Data volumes quickly go beyond our comprehension but results of statistical analysis of pattern recognition do not replace human reasoning (algorithms and machines do not “think” -- at least so far). Furthermore, there is a severe misconception that computational predictions might demonstrate or “validate” the bioactivity of compounds. Notably, these and other misunderstandings may not be evident to researchers and students who are just beginning to use computational methods. We encourage the community to avoid judging a computational research project to be “incomplete” because it does not include experiments or to be “complete” just because it incorporates many different computational methods. The question of completeness is not separable from scientific rigor and adequate conduct of methodologies, be they computational or experimental in nature. Furthermore, let us not consider a computational analysis as a “luxury item” to decorate a project report, grant application, or scientific paper with “pretty pictures”.

## Using methods for the right reasons

Mainstream media usually disseminate inaccurate or exaggerated reports about the capabilities of computational methods without also mentioning their limitations and flaws. Simultaneously, mass job search engines commonly offer job opportunities with extensive lists of different computational tools as requirements. These factors, among others, continuously put pressure on researchers to increase their productivity and academic credentials to further their careers at the expense of scientific rigor and the quality of research. CADD is not the exception of the increasing trend that disrupts the traditional academic structure in favor of a more market-oriented approach. One of the consequences of this phenomenon is that many young professionals and new CADD practitioners direct their efforts to increase the volume of their curriculum vitae rather than using CADD methods to answer relevant scientific questions.

The popularity of computational methods or tools often jeopardizes rational selection. Newcomers often turn to frequently used methods that are well-validated. However, the justification is questionable if the technique is merely used because it is “popular” (e.g., “follow the crowd” because “it should be right.”). Without properly addressing the question at hand, computational analysis applying irrelevant approaches is misleading or propagates errors. Arguably, one of the most misused guidelines in drug discovery is the Lipinski Rule of Five, ^15^ which is often confused with assessing “drug-likeness”. Another common pitfall among newcomers to CADD is using docking to predict “real” protein-ligand complexes, given its popularity and easy-of-use. Practitioners should use methods for the right reasons and not just because everybody else is using them. This requires knowledge of underlying theories and sound scientific judgment.

## General recommendations for the proper use of CADD resources

In the authors’ opinion, the following recommendations should be helpful to CADD novices and multidisciplinary research teams attempting or planning to use computational approaches to guide drug discovery projects. Similar to Table 1, the list is not exhaustive, but is also intended to stimulate an open discussion within the scientific community.

• Intense study of the literature is essential to acquire knowledge. Like experimental techniques, also CADD methods require proper training to become familiar with their applicability domains, approximations and limitations.
• Computational research projects should primarily be problem-oriented rather than technique-oriented, unless the development of new techniques themselves is the focus of the problem to be addressed. Projects (including dissertations) should be well-structured according to scientific criteria or milestones, but by no means represent a compilation or aggregated use of techniques applied to the same data. Before deciding which computational approaches and tools to use, a comprehensive research of the literature should be conducted and exemplary applications should be reviewed. Then, based on the experimental information available, appropriate computational methods and strategies should be applied. Rushing into calculations with software packages, even with excitement, is typically detrimental if the applied methods are not scientifically justified. In addition to researching methodological aspects, it is also mandatory to carefully review the available experimental findings. For example, prior to applying virtual screening techniques to search for new compounds with activity against high-profile and extensively explored targets, care should be taken not to overlook prior art in the field and avoid engaging in scientifically naive computational efforts. One should avoid setting the goals of a drug discovery project relative to a technique by pursuing a “tool-oriented approach”. Instead, planning computational components of an interdisciplinary research project should focus on the ultimate scientific goals. Students should realize and keep in mind that learning and applying different techniques across disciplines is desirable, but they should be used in harmony to answer research questions.
• Seek supervision or advice from experts and do not hesitate to ask. Consultation prior to engaging in a new scientific adventure will not only save time and resources but also help to plan a scientifically sound approach.
• Avoid excessive use of buzzwords such as “artificial intelligence” or “machine learning” when they are not applicable, which contributes to inappropriate hype associated with computational methods. For example, there is no need to use the AI or “machine intelligence” label for compound classification methods that are already applied for decades.
• Keep in mind that many theoretical disciplines contribute to CADD, which have a long history on their own such as machine learning.
• As in any wet lab experiment, input data quality is of critical importance for the outcome of computational studies. Awareness of data curation requirements is essential for the integrity of computational work.
• The uncritical or uneducated use of web-accessible computational tools or servers to generate new compounds, calculate molecular properties, or predict target structures and protein-ligand complexes is a major source of errors propagating through interdisciplinary projects.

## Concluding remarks

The current pandemic and related funding constraints in some countries and institutions have motivated many researchers at different levels to redirect their efforts from difficult to sustain experimental studies to easy-to-use computational tools that can be employed remotely. Also, reviewer panels of many current grant applications from academia, non-for profit, or the industry currently tend to give priority to research proposals that involve AI. Although this contributes to the popularity of CADD, it also comes at a cost. If uneducated CADD studies enter the realm of science fiction harm is done to this field, its credibility and acceptance, and further scientific development. This must be avoided at all costs. The methods used in CADD should not be applied as black boxes, which can be enabled by just a few hours of hands-on experience such as provided in workshops. Without sufficient understanding of the scope, complexity, and theoretical foundations of these computational methodologies such efforts will inevitably fail and discredit investigators and their work as well as the field as a whole. Newcomers to the area, including students, early-career scientists, and seasoned investigators attempting to re-focus their efforts should be fully aware that, similar to experiments, profound knowledge of CADD concepts and informed use of CADD tools is a must. A simple yet fundamentally important rule applies: “Don’t compute what you don’t understand”. In addition to the general recommendations outlined in this *Opinion*, we wish to encourage students, newcomers, and practitioners of CADD to use the computational tools and resources for the right reasons, not just because they are easily accessible. Similarly, we highly encourage the scientific community to avoid applying computational methods just because they are popular. Instead, it is strongly recommended to identify scientific questions that can be addressed appropriately using CADD approaches - and avoid others where computational efforts become questionable. In general, computational studies that cannot be reported in established peer-reviewed journals whose scope includes CADD are to be considered with appropriate caution, both by experts and novices to the field. This also applies to the use of modeling web servers. While the integrity of publicly accessible computational tools can be guaranteed by the developers, addressing ill-defined questions or tasks using these tools is beyond their control. Recognizing the benefits of the open post-publication review culture of *F1000Research*, we would be delighted if this contribution would catalyze open discussions among readers to raise further awareness of latent problematic issues in the CADD area and support its further scientific development.

## Data availability

No data is associated with this article.
