# Supplementary material for: Rationality over fashion and hype in drug design
Source: F1000Res. 2021 May 18;10:Chem Inf Sci-397. [Version 1] doi: 10.12688/f1000research.52676.1 (PMC8201421; doi:10.12688/f1000research.52676.1)
Supplement: Peer review history

# Peer review: Reviewer response for version 1

**DOI:** 10.5256/f1000research.55986.r86331

**Published:** 2021-06-10

**Authors:** Maria Sorokina

**Affiliations:** 1 Institute for Inorganic and Analytical Chemistry, Friedrich-Schiller University Jena, Jena, Germany

The authors discuss the dangers of the growing trend of using computer assisted drug discovery tools without a proper training nor understanding of the fundamental concepts laying behind such approaches. The topic is indeed very important to discuss, and the past year of working away from the bench and the tremendous amount of publications produced using, often not totally correctly, CADD and other bioinformatic tools and the examples illustrated in Table 1, emphasize this need of reminding that computational tools, although easy to use, still require understanding of the concepts they are built on.

 Regarding the sentence ‘Avoid excessive use of buzzwords such as “artificial intelligence” or “machine learning” when they are not applicable, which contributes to inappropriate hype associated with computational methods', I would also add to avoid the usage of “deep learning” another very trendy buzzword, too often misused.

 I thank the authors for this article, as such topics are extremely important to be voiced out.

Is the topic of the opinion article discussed accurately in the context of the current literature?

Yes

Are arguments sufficiently supported by evidence from the published literature?

Yes

Are all factual statements correct and adequately supported by citations?

Yes

Are the conclusions drawn balanced and justified on the basis of the presented arguments?

Yes

Reviewer Expertise:

NA

I confirm that I have read this submission and believe that I have an appropriate level of expertise to confirm that it is of an acceptable scientific standard.

# Peer review: Reviewer response for version 1

**DOI:** 10.5256/f1000research.55986.r85580

**Published:** 2021-05-19

**Authors:** Bruno Villoutreix

**Affiliations:** 1 l’Institut national de la santé et de la recherche médicale (INSERM), Paris, France

This review about fashion and hype in the field of drug design, written by experts, is indeed very timely and of high interest. The observation applies to many fields related to medicine and biology, to most technologies and even research topics.

 This opinion paper should be read by politicians, decision makers, journalists, students, the citizens not involved in research and many scientists. Even more so, it should be read by people defining guidelines for grant applications and by investors that are ready to put millions on a new “in silico” technology that is going to save the world but that has never been published and is only documented on some nice brochures full of buzz words (the so-called proprietary tools that nobody can try or evaluate).

 Indeed, nowadays, if a grant or a research paper does not use every two sentences words like game-changer, disruptive, AI, big data, multi-scale, large scale, ultra-large something or quantum something,  it has little chance to succeed (in my opinion “quantum whatever” is going to be the next hype after AI, and if you have both,  AI + quantum computing or quantum scoring or quantum ADMET or quantum digital twin, then you win the lottery, meaning grants, investors’ money, promotion, medals, prize, fame, and the person will definitively go on TV, get numerous "like" in social networks and become a scientific star invited by politicians to guide the remaining ignorant scientists that do not buy buzz words…).

 The complexity is that, in some cases, these methods are going to help, in some others, this is the wind blowing in the forest. Experts in the field know but they are not invited to comment or, if they are, they do not say anything because they are afraid to be considered old school. The reality is of course very different, no problem being enthusiastic about some approaches, about developing "user-friendly" methods, about testing new concepts, but global brainwashing, overselling and propaganda about AI and related are damaging research and in the field of health, these promises will hurt patients, and confuse even more people.

 How to stop this given the millions of dollars behind, given global mass brainwashing and the fact that most humans prefer fairy tales than truth? I do not know.

 Definitively the present opinion paper can help, the one difficulty I see is that people who should read it will not do so and worse, do not want to hear about such discussion as it may kill their business plan or chance of getting famous. Maybe, if the scientific community that refuses “fashion research” starts to make noise, some changes will occur.

Is the topic of the opinion article discussed accurately in the context of the current literature?

Yes

Are arguments sufficiently supported by evidence from the published literature?

Yes

Are all factual statements correct and adequately supported by citations?

Yes

Are the conclusions drawn balanced and justified on the basis of the presented arguments?

Yes

Reviewer Expertise:

drug discovery, structural bioinformatics, chemoinformatics, molecular medicine

I confirm that I have read this submission and believe that I have an appropriate level of expertise to confirm that it is of an acceptable scientific standard.
